# Evidence for decreased copper associated with demyelination in the corpus callosum of cuprizone-treated mice

**DOI:** 10.1093/mtomcs/mfad072

**Published:** 2024-01-04

**Authors:** James B W Hilton, Kai Kysenius, Jeffrey R Liddell, Stephen W Mercer, Dominic J Hare, Gojko Buncic, Bence Paul, YouJia Wang, Simon S Murray, Trevor J Kilpatrick, Anthony R White, Paul S Donnelly, Peter J Crouch

**Affiliations:** Department of Anatomy & Physiology, The University of Melbourne, Victoria 3010, Australia; Department of Anatomy & Physiology, The University of Melbourne, Victoria 3010, Australia; Florey Institute of Neuroscience and Mental Health, The University of Melbourne, Victoria 3010, Australia; Department of Anatomy & Physiology, The University of Melbourne, Victoria 3010, Australia; Department of Anatomy & Physiology, The University of Melbourne, Victoria 3010, Australia; Atomic Medicine Initiative, University of Technology Sydney, Sydney, New South Wales 2007, Australia; School of Chemistry and Bio21 Molecular Science and Biotechnology Institute, The University of Melbourne, Victoria 3010, Australia; School of Earth Sciences, The University of Melbourne, Victoria 3010, Australia; Department of Anatomy & Physiology, The University of Melbourne, Victoria 3010, Australia; Department of Anatomy & Physiology, The University of Melbourne, Victoria 3010, Australia; Florey Institute of Neuroscience and Mental Health, The University of Melbourne, Victoria 3010, Australia; Department of Anatomy & Physiology, The University of Melbourne, Victoria 3010, Australia; Florey Institute of Neuroscience and Mental Health, The University of Melbourne, Victoria 3010, Australia; Queensland Institute of Medical Research Berghofer, Herston, Queensland 4006, Australia; School of Chemistry and Bio21 Molecular Science and Biotechnology Institute, The University of Melbourne, Victoria 3010, Australia; Department of Anatomy & Physiology, The University of Melbourne, Victoria 3010, Australia

**Keywords:** copper, corpus callosum, cuprizone, multiple sclerosis, demyelination, Cu(atsm), myelin, protection, therapy

## Abstract

Demyelination within the central nervous system (CNS) is a significant feature of debilitating neurological diseases such as multiple sclerosis and administering the copper-selective chelatorcuprizone to mice is widely used to model demyelination *in vivo.* Conspicuous demyelination within the corpus callosum is generally attributed to cuprizone's ability to restrict copper availability in this vulnerable brain region. However, the small number of studies that have assessed copper in brain tissue from cuprizone-treated mice have produced seemingly conflicting outcomes, leaving the role of CNS copper availability in demyelination unresolved. Herein we describe our assessment of copper concentrations in brain samples from mice treated with cuprizone for 40 d. Importantly, we applied an inductively coupled plasma mass spectrometry methodology that enabled assessment of copper partitioned into soluble and insoluble fractions within distinct brain regions, including the corpus callosum. Our results show that cuprizone-induced demyelination in the corpus callosum was associated with decreased soluble copper in this brain region. Insoluble copper in the corpus callosum was unaffected, as were pools of soluble and insoluble copper in other brain regions. Treatment with the blood–brain barrier permeant copper compound Cu^II^(atsm) increased brain copper levels and this was most pronounced in the soluble fraction of the corpus callosum. This effect was associated with significant mitigation of cuprizone-induced demyelination. These results provide support for the involvement of decreased CNS copper availability in demyelination in the cuprizone model. Relevance to human demyelinating disease is discussed.

## Graphical Abstract

**Graphical Abstract:**
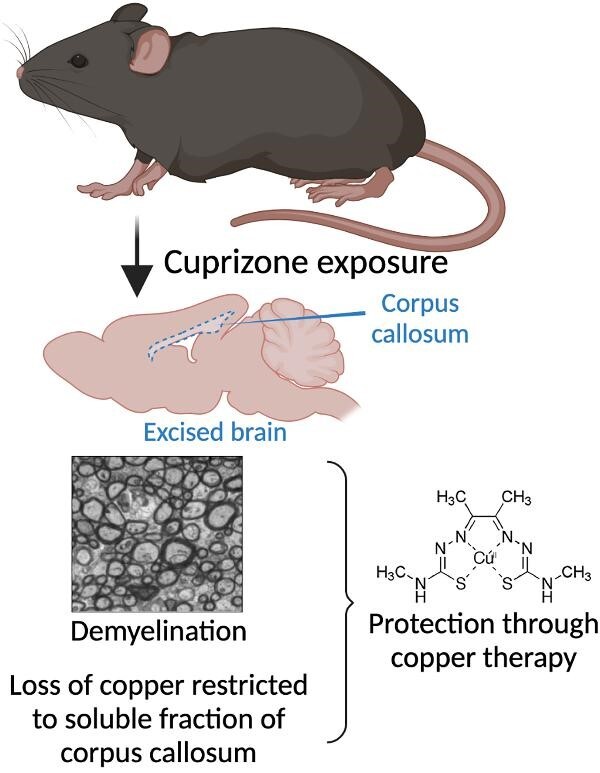
Cuprizone-induced demyelination in the mouse corpus callosum involves depletion of copper in the soluble fraction of this vulnerable brain region. Pharmacological copper delivery to the brain is protective.

## Introduction

Dietary acquisition of copper is essential because of its requirement in diverse cuproenzymes tasked with critical biological functions throughout the body.^1^ Significance of this is illustrated by Menkes disease where mutations affecting the copper transporter ATP7A result in severe impairment of copper trafficking leading to restricted copper availability, neurological dysfunction, and death of afflicted individuals within the first 3 yr of life.^2^ Despite ubiquitous expression, milder mutations to the same transporter selectively affect the central nervous system (CNS) to cause a rare form of motor neuropathy.^3,4^ Selective vulnerability of the CNS relates to natural copper turnover rates and uptake. Copper turnover is exceedingly slow in the CNS and becomes even slower in conditions of limited copper availability^5^ and ubiquitous heterozygosity for the copper uptake transporter Ctr1 (*Slc31a1*) causes more pronounced copper deficiency in the brain when compared to other organs.^6^ Thus, the CNS is particularly ill-equipped to accommodate changes affecting copper availability.

It is the loss of axonal myelination that gives rise to the neurological deficits that manifest in conditions such as multiple sclerosis^7^ and separate lines of evidence indicate that disrupted copper availability within the CNS affects the axonal myelination that is required for optimal propagation of inter-neuronal signal transfer. Not least of which, insufficient copper availability affecting myelination in humans has been documented in the form of copper deficiency myelopathy,^8^ a disorder that manifests as neurological symptoms associated with demyelination, white matter lesions, and axonopathy. Furthermore, overzealous treatment of the copper accumulation condition of Wilson's disease is reported to cause widespread CNS demyelination associated with hypocupremia.^9^

Modeling demyelination *in vivo* provides opportunity to elucidate disease mechanisms and assess putative therapeutic targets. To this end, treating animals with cuprizone appears to provide an experimental model that connects CNS demyelination to copper availability. Cuprizone is a copper-selective chelator and when added to the diet of various animal species induces demyelination within specific brain regions.^10–12^ Demyelination induced by cuprizone is attributed to toxicity toward myelin-generating oligodendrocytes as remyelination subsequent to removal of cuprizone involves proliferation of oligodendrocyte progenitor cells.^13^ Moreover, the demyelinating toxicity of cuprizone is generally attributed to depletion of available copper in the CNS.^14^ It is notable, therefore, that clear evidence for cuprizone-induced changes in CNS copper associated with demyelination is yet to be reported. Here we have addressed this topic by analysing brain tissue samples from cuprizone-treated mice using an inductively coupled plasma mass spectrometry (ICPMS) methodology that enables measurement of copper in low volume samples.^15^ Additionally, we assessed potential to remedy cuprizone-induced demyelination using the blood–brain barrier permeant copper-delivery agent Cu^II^(atsm).^16^ We focused on demyelination within the corpus callosum in the brains of C57BL/6 mice treated with cuprizone at 0.2% (w/w) dietary supplement for 40 d as this approximates the most used experimental paradigm.

## Methods

### Study design

**Fig. 1:**
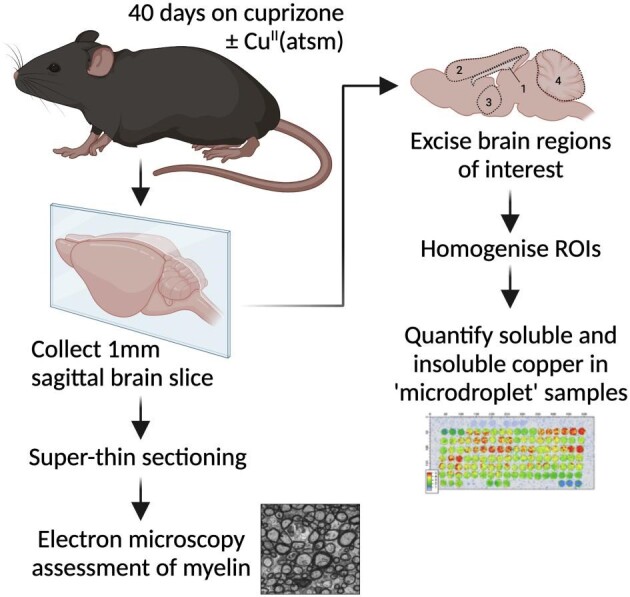
Study design. Male and female C57BL/6 mice at 8 wk old were treated with cuprizone for 40 d by supplementing their standard chow. Cu^II^(atsm) was administered to a cohort of mice concurrently with cuprizone by daily gavage. Control mice were provided standard chow. Brain regions of interest (ROIs) for copper analyses were: 1, corpus callosum; 2, cerebral cortex; 3, thalamus/hypothalamus; and 4, cerebellum.

Mice were treated with cuprizone prior to collecting brain samples for assessment of myelination or copper partitioned into soluble and insoluble fractions (Fig. 1). Myelination was assessed by electron microscopy. Copper levels were assessed using an ICPMS “microdroplet” methodology.^15^ Control mice were fed standard chow whereas cuprizone-treated mice were fed standard chow supplemented with cuprizone at 0.2% (w/w). Additional mice were also treated with the blood–brain barrier permeant copper-delivery agent Cu^II^(atsm).

### Study approval

All research involving mice was approved by a University of Melbourne Animal Experimentation Committee (approval numbers 1513554 and 1613995) and conformed with guidelines of the Australian National Health and Medical Research Council.

### Mouse treatments

Male and female C57BL/6 mice were randomly allocated into four treatment groups: control, cuprizone, control plus Cu^II^(atsm), or cuprizone plus Cu^II^(atsm). Control mice received powdered feed. Mice treated with cuprizone received powdered feed supplemented with 0.2% (w/w) cuprizone (Sigma). All powdered feed was refreshed daily.

Cu^II^(atsm) was synthesized as described previously^17,18^ and prior to administration to mice a fine suspension at 7.5 mg ml^−1^ was prepared in standard suspension vehicle (SSV; 0.9% w/v NaCl, 0.5% w/v Na-carboxymethylcellulose, 0.5% v/v benzyl alcohol, 0.4% v/v Tween 80) each day via sonication. Mice receiving Cu^II^(atsm) were gavaged with Cu^II^(atsm) daily at 30 mg kg^−1^ body weight. All other mice were sham gavaged with an equivalent volume of SSV that did not contain Cu^II^(atsm). Treatment with Cu^II^(atsm) and cuprizone commenced when the mice were 8 wk old and continued for 40 d.

### Tissue collection from mice

At the end of the treatment period mice were deeply anaesthetized by intraperitoneal injection of saline solution supplemented with ketamine and xylazine (120 and 16 mg kg^−1^ body weight, respectively) then transcardially perfused with phosphate-buffered saline supplemented with phosphatase inhibitors (Phosphatase Inhibitor Cocktail 2; Sigma), protease inhibitors (Complete EDTA-free Tablets; Roche), and heparin (20 U ml^−1^). After perfusion, mice were dissected to remove brains and the collected tissue either snap frozen and stored at −80°C (left hemisphere) or fixed in glutaraldehyde for subsequent histological examination (right hemisphere).

### Tissue processing for copper analyses

Frozen brain hemispheres were used to produce 1-mm-thick sagittal sections. These were then macroscopically dissected to produce regions enriched for corpus callosum (whole corpus callosum), cerebral cortex, cerebellum, and thalamus/hypothalamus (Fig. 1). These samples were homogenized using polypropylene pestles in tris-buffered saline (TBS) supplemented with 0.5% (v/v) phosphatase inhibitor cocktail 2 (Sigma), 2% (w/v) Complete EDTA-free protease inhibitor (Roche), and 5% (v/v) DNase. Homogenates were then separated into TBS-soluble fractions and TBS-insoluble pellets by centrifugation (21 000 RCF, 4°C) for 30 min. Insoluble pellets were resuspended in the same TBS-based homogenization buffer and re-homogenized using polypropylene pestles to produce TBS-insoluble suspensions. Protein content of TBS-soluble and -insoluble fractions was determined using the BCA Assay (Thermo Fisher Scientific), then all samples were normalized to a consistent protein concentration using the TBS homogenizing buffer described earlier.

Western blot analysis was used to assess effectiveness of the TBS-soluble/TBS-insoluble fractionation process. Briefly, whole brain tissue from a control mouse was fractionated as described earlier then analysed using western blot procedures previously described.^19^ Primary antibodies used for detecting the following proteins of interest were as follows: Superoxide dismutase 1 (Sod1, Abcam ab16831); glyceraldehyde 3-phosphate dehydrogenase (Gapdh, Cell Signalling 2118); β-actin (Cell Signalling 4970); 2ʹ3ʹ-cyclic nucleotide 3ʹ-phosphodiesterase (Cnp, Abcam ab6319); myelin oligodendrocyte glycoprotein (Mog, Abcamab 109746); and histone H3 (Cell Signalling 9715).

### Laser ablation ICPMS

To measure levels of copper partitioned into soluble and insoluble fractions we utilized the laser ablation-ICPMS based microdroplet method described previously.^15^ In brief, 0.5 μl aliquots of TBS-soluble and insoluble material were loaded onto microscope slides. Equivalent volumes of TBS homogenizing buffer were loaded onto the slides as matrix controls and standards consisting of known copper concentrations made up in TBS homogenizing buffer were also loaded. All sample slides were air-dried overnight then imaged and analysed using laser ablation-ICPMS.

### Microscopy

Brain samples collected from mice at the end of the 40 d treatment period were cut sagittally into 1 mm slices and fixed in glutaraldehyde buffer (2.5% glutaraldehyde, 2% PFA, 0.1 M cacodylate buffer) overnight at 4°C. Tissues were then rinsed three times with 100 mM cacodylate buffer, incubated in 1% osmium tetroxide and 1.5% potassium ferrocyanide in distilled water for 2 h, then rinsed with distilled water and stored overnight at 4°C. Tissues were next dehydrated through a series of ethanol and acetone, followed by infiltration and embedding in Spurr's resin and polymerized overnight at 70°C.

To examine axonal diameter and myelin sheath thickness in the corpus callosum (rostral), ultrathin sections (90 nm) were stained with uranyl acetate and lead citrate and examined by transmission electron microscopy (JEOL1101, Inc., USA). Electron microscope images were taken with a Megaview III FW (Olympus Soft Imaging Solutions, Münster, Germany) camera equipped to the microscope. Images were quantified using ImageJ (http://rsbweb.nih.gov/ij/) with myelin thickness and axonal diameter determined by measuring total outer diameter (*d*_0_; inclusive of myelin sheath) and inner diameter (*d_i_*; exclusive of myelin sheath), with measurements performed on two perpendicular planes per axon and the average per axon used in final analyses. An average of 115 axons was counted per animal.

### Statistical analyses

All statistical analyses were performed using GraphPad Prism Version 9. Prior to performing statistical analyses to compare mean differences between groups, data were assessed for outliers using the ROUT20 (robust regression followed by outlier identification) method^20^ and identified outliers excluded from further analysis. Statistical significance of observed differences between group means was assessed using the two-tailed Student's *t*-test or ordinary one-way ANOVA with Šídák's multiple comparisons test. Statistical significance was determined as *P* < 0.05.

## Results and discussion

**Fig. 2:**
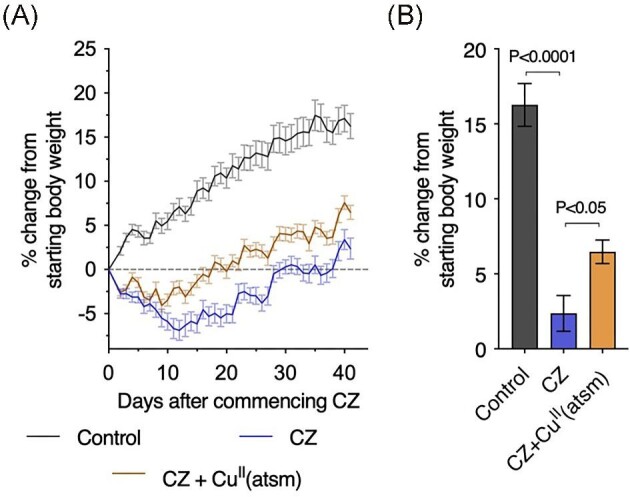
Body weight changes in mice treated with cuprizone (CZ).(**A**) Daily body weight recordings.(**B**) Body weight results at the end of the 40 d study period. All body weight data are expressed as percentage change from starting body weight to accommodate inherent differences between male and female mice. Each treatment group had a 1:1 female: male allocation. Results represent mean values (±S.E.M.) for 30–40 mice per treatment group. *P*-values indicate significant differences.

Supplementing the food of mice with cuprizone is widely used to induce demyelination in the brain, particularly within the corpus callosum.^10,11^ These myelination effects are associated with significant decreases in body weight while the animals are maintained on the cuprizone-supplemented diet, generally displaying an initial decline that is followed by a subsequent steady increase in body weight that approximates that of animals on a regular diet.^21,22^ Mice included in our study reproduced these body weight changes (Fig. 2A). Oral co-treatment with the blood–brain barrier permeant bis(thiosemicarbazone)-copper complex Cu^II^(atsm)^23^ mitigated these cuprizone-induced changes in body weight, resulting in a significantly higher body weight at the end of the 40 d study period when compared to mice on cuprizone that were sham treated with the vehicle for Cu^II^(atsm) (Fig. 2B).

**Fig. 3:**
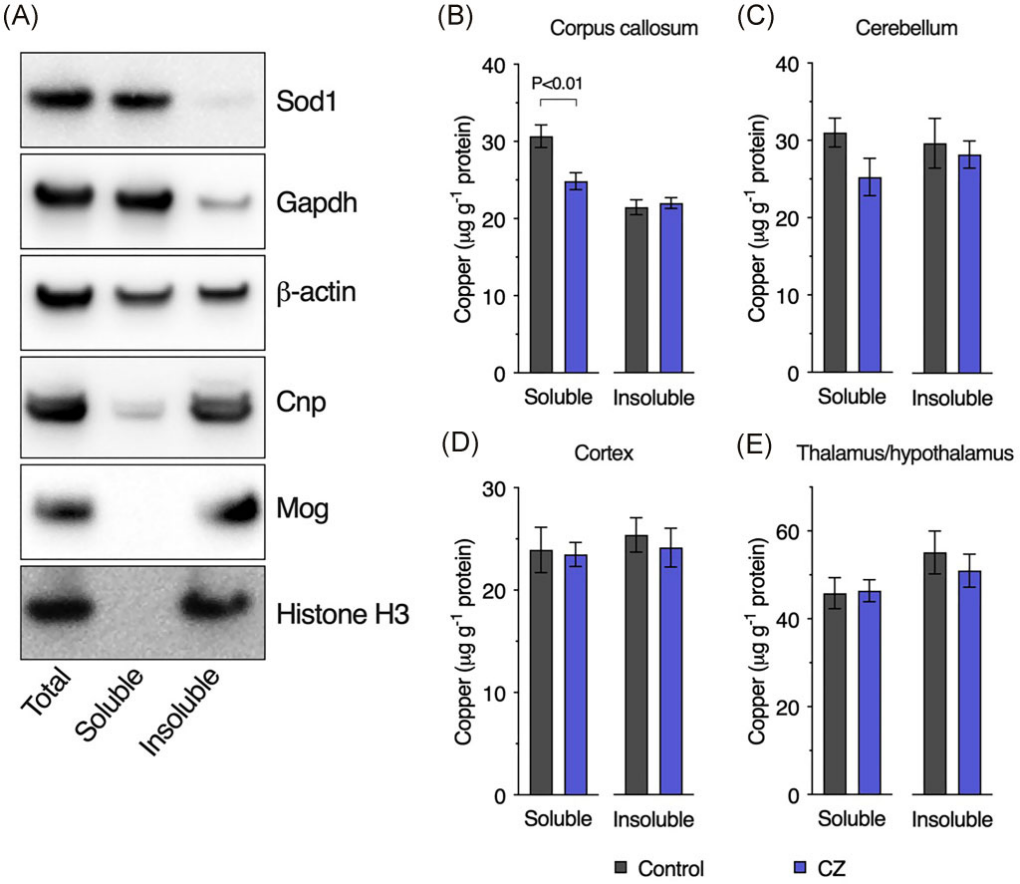
Copper concentrations in brain regions from cuprizone-treated mice. Brain tissue was separated into tris-buffered saline (TBS)-soluble and TBS-insoluble fractions. Western blot analyses (**A**) illustrate the effect of this fractionation as follows: cytosolic protein superoxide dismutase 1 (Sod1); cytosolic protein glyceraldehyde 3-phosphate dehydrogenase (Gapdh); ubiquitous protein β-actin; cytoskeletal and plasma membrane protein (oligodendrocytes) 2ʹ3ʹ-cyclic nucleotide 3ʹ-phosphodiesterase (Cnp); plasma membrane protein (oligodendrocytes) myelin oligodendrocyte glycoprotein (Mog); and nuclear protein histone H3.Copper concentrations in TBS-soluble and TBS-insoluble extracts from (**B**) corpus callosum, (**C**) cerebellum, (**D**) cortex, and (**E**) thalamus/hypothalamus from control and cuprizone (CZ) treated mice. Results represent mean values (±S.E.M.) for 6–10 mice per treatment/brain region. *P*-value indicates significant difference.

To examine the impact of cuprizone on brain copper concentrations we utilized a microdroplet methodology developed for laser ablation-ICPMS quantitation of endogenous copper.^15^ This experimental approach enabled two key advancements. First, homogenization in a TBS-based buffer enabled segregation of endogenous copper into soluble and insoluble pools. Although a relatively crude biochemical fractionation, this nonetheless enabled some discrimination between copper associated with cytosolic components (soluble fraction) from copper associated with other cellular components such as the nucleus and plasma membrane (insoluble fraction). Segregation of distinct proteins relative to the TBS-soluble and TBS-insoluble fractions illustrates effectiveness of the fractionation process (Fig. 3A). Second, suitability of the method for quantifying copper in low volume samples enabled assessment of copper specifically within the corpus callosum despite its small anatomical size.

**Fig. 4:**
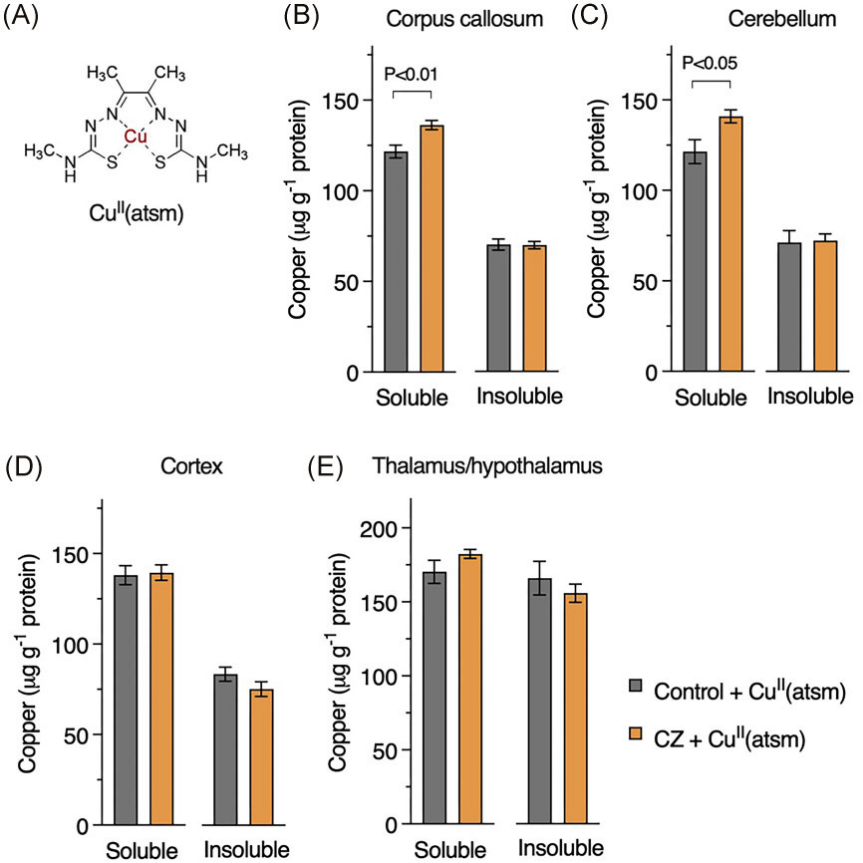
Copper concentrations brain regions of mice after treatment with Cu^II^(atsm) and relative to treatment with cuprizone (CZ). (**A**) Chemical structure of Cu^II^(atsm) with central copper atom highlighted. Partitioning of copper into tris buffered saline (TBS)-soluble and TBS-insoluble fractions in (**B**) corpus callosum, (**C**) cerebellum, (**D**) cortex, and (**E**) thalamus/hypothalamus brain regions. Results represent mean values (±S.E.M.) for 9–10 mice per treatment/brain region. *P*-values indicate significant differences.

Despite systemic exposure to cuprizone, the impact on brain copper was restricted to the TBS-soluble fraction of the corpus callosum (Fig. 3B–E). Soluble copper in the cerebellum trended toward a similar decrease, but this result was not statistically significant (*P* = 0.085). Oral co-treatment with Cu^II^(atsm) (Fig. 4A) increased brain copper levels, where higher responses to the treatment in the TBS-soluble fraction of the corpus callosum and cerebellum indicated some selectivity with respect to how the cuprizone-treated mouse brain responds to Cu^II^(atsm) (Fig. 4B–E). Brain region specific responses to Cu^II^(atsm) are consistent with clinical outcomes for Cu^II^(atsm) when used as a PET tracer for the neurological conditions of amyotrophic lateral sclerosis, Parkinson's disease, MELAS, and Alzheimer's disease.^24–27^

**Fig. 5:**
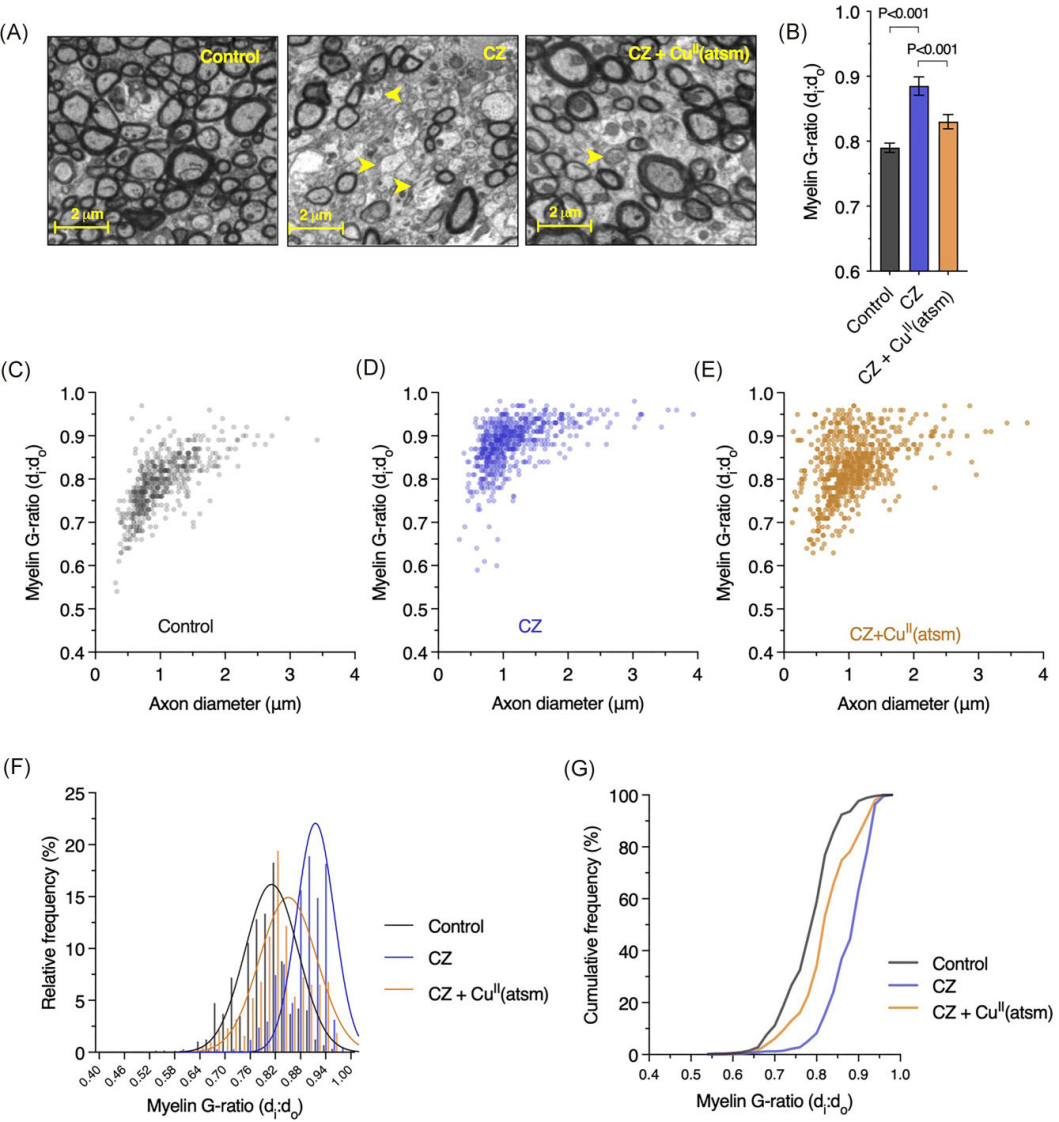
Treatment with Cu^II^(atsm) remedies cuprizone (CZ)-induced demyelination in the corpus callosum. (**A**) Representative electron microscopy images for myelination of axons in corpus callosum of control and cuprizone-treated mice, and mice on cuprizone treated with Cu^II^(atsm). Yellow arrow heads highlight axons with low level myelination. (**B**) Myelination of axons in corpus callosum expressed as G-ratio, where higher values indicate demyelination. Results represent mean values (±S.E.M.). *P*-values indicate significant differences. (**C**–**E**) Myelin G-ratio relative to axon diameter in corpus callosum of control and cuprizone model mice, and cuprizone model mice treated with Cu^II^(atsm). Dots in scatter plots show the number of individual axonal measurements recorded. (**F, G**) Myelin G-ratio in corpus callosum of control and cuprizone model mice, and cuprizone model mice treated with Cu^II^(atsm) expressed as relative and cumulative frequencies. Fitted curves in (**F**) show Gaussian least squares fit. All data derived from 5–6 mice per treatment group with an average of 115 axons counted per animal.

**Fig. 6:**
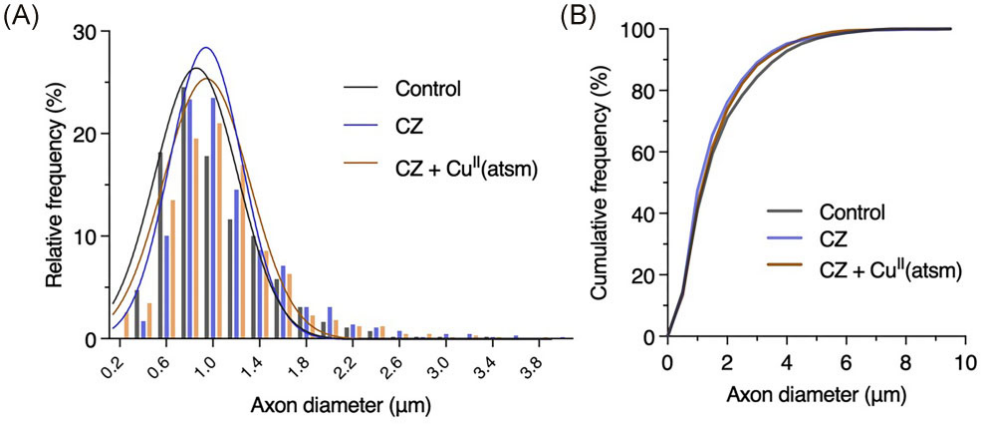
Treatment with Cu^II^(atsm) does not affect axonal diameter. Axonal diameter in corpus callosum of control and cuprizone (CZ) treated mice, and cuprizone mice treated with Cu^II^(atsm) expressed as (**A**) relative and (**B**) cumulative frequencies. Fitted curves in **a** show Gaussian least squares fit. All data derived from 5–6 mice per treatment group with an average of 115 axons counted per animal.

In line with previous reports,^12^ demyelination was evident within the corpus callosum of mice on cuprizone as represented by decreased myelin sheath thickness relative to axonal diameter resulting in an increased G-ratio (Fig. 5). This response to cuprizone was ameliorated by treatment with Cu^II^(atsm) as seen by maintenance of near-physiological myelination. This response was driven by improvements in myelination as changes in axonal diameter were not evident (Fig. 6).

Disrupted availability of copper is implicated in human afflictions that involve demyelination in the CNS,^8,28,29^ including the severe copper deficiency condition of Menkes disease.^30–33^ A causal role for copper availability in demyelination is supported by studies that show repeated administration of chelating agents to rabbits resulted in decreased serum copper levels and spinal cord demyelination^34^ and that intergenerational dietary copper restriction caused CNS demyelination in rats.^35,36^ Notably, the latter could be corrected in the F_2_ generation if pregnant dams were provided a copper-supplemented diet or if the neonate pups were transferred to a copper-replete foster mother.^37^

Consistent with this connection between decreased circulating/dietary copper and impaired CNS myelination, a commonly used mammalian model of CNS demyelination involves administration of the copper-chelating compound cuprizone to mice.^10–12^ But despite being broadly described as “inducing CNS demyelination by feeding with the copper chelator cuprizone,” reports on copper being involved in CNS demyelination in the cuprizone model are inconclusive. In one short-duration study in which cuprizone was fed to mice for 1 wk and failed to induce demyelination, the copper concentration in the whole homogenized brain was reported as increased.^38^ Similar accumulation of copper in the brain was also found in a long-term cuprizone study in which mice were treated with the compound at 0.2% (w/v) in drinking water for 3–9 mo, with unquantified demyelination reported to be associated with increased copper concentrations in the forebrain/midbrain, cerebellum and pons-medulla.^39^

Differentiated from these studies, Venturini *et al*.^40^ reported that copper concentrations in the brain were decreased approximately 40% after treating with cuprizone for 2 wk but were unchanged in animals treated with a pre-existing cuprizone–copper complex. Commensurate decreases in the copper-dependent activities of monoamine oxidase and cytochrome c oxidase were reported for the cuprizone only group^40^ indicating restricted delivery of copper to these cuproenzymes. However, this study only assessed whole brain homogenates and again did not provide evidence for demyelination or copper levels within the affected corpus callosum. Other reports indicate that treatment with cuprizone has no effect on brain copper levels. In the absence of evidence for demyelination, Jeyasingham *et al*.^41^ reported no change in total brain copper after treating with 0.5% cuprizone for 7 wk, and in the absence of quantified data for demyelination, Moldovan *et al*.^42^ reported no change in copper in the cerebellum, hippocampus, thalamus, striatum or cortex of mice treated with cuprizone at 0.2% for 6 wk.

Most recently, Morgan *et al*.^43^ examined the relationship between copper availability and CNS demyelination in mice via a range of experimental paradigms, including treatment with cuprizone, treatment with other copper-selective chelators (i.e. neocuproine and D-penicillamine), dietary copper supplementation using CuSO_4_, and various combinations thereof. While cuprizone robustly induced demyelination in the brain, the other chelators did not. Treatment with D-penicillamine induced clinical features of tail flaccidity and hindlimb paralysis and weakness, all of which are features associated with spinal cord demyelination in the experimental autoimmune encephalomyelitis model of MS.^44^ Spinal cord pathology was reported as not detected in the D-penicillamine treated mice (data not shown) and copper supplementation using CuSO_4_ corrected all D-penicillamine-induced clinical features.^43^ By contrast, copper supplementation using CuSO_4_ did not protect against cuprizone-induced demyelination in the brain. Outcomes from the broad range of complementary experimental paradigms, including rescue from cuprizone-induced demyelination by the additional administration of D-penicillamine, led to the interpretation that cuprizone may cause demyelination in the brain through formation of a toxic cuprizone–copper complex and the conclusion that “the well-known effects of the demyelinating agent cuprizone are not due to depletion of copper as might be expected.”^43^ The underpinning logic here is that exposure to a second copper-binding agent would not have a protective effect if the demyelinating mechanism of action for cuprizone involved copper depletion. It is notable, therefore, that amongst all existing reports on copper in brain samples collected from mice treated with cuprizone, including that of Morgan *et al*.,^43^ none provides data for copper within the corpus callosum, the most reported site of demyelination in the cuprizone model.^10,11^ The absence of copper data for the corpus callosum likely reflects limited tissue availability and this is a limitation that is overcome by our analytical method.^15^ Our data show that cuprizone does decrease copper within the corpus callosum.

Less copper within the corpus callosum would seem incompatible with the formation of a toxic cuprizone–copper complex. However, a possible alternate interpretation here is that rather than cuprizone causing demyelination by depleting copper, the observed loss of copper from the corpus callosum is the result of a toxic cuprizone–copper complex causing degradation and clearance of copper-containing structures such as myelin. Our result for copper loss being evident in the TBS-soluble fraction yet myelin associated proteins Cnp and Mog being enriched in the TBS-insoluble fraction (Fig. 3) suggests that if a toxic cuprizone–copper complex does cause loss of copper-containing myelin structures, these structures are not total myelin per se and that the impact of cuprizone on copper-containing myelin structures is restricted to those that are extractable in an aqueous solution such as TBS. Further work is clearly required to elucidate the relationship between copper, cuprizone and demyelination *in vivo*, especially while this model of CNS demyelination continues to be used to elucidate mechanisms of disease and develop new therapeutics for significant diseases such as multiple sclerosis. We contend that measurement of copper is essential if conclusions are to be drawn on the effects of cuprizone related to copper availability and/or copper-mediated toxicity. Assessment of copper in brain samples from cuprizone treated mice has to date only measured total copper and has insufficiently explored the possibility that cuprizone-induced demyelination involves mechanisms in which copper allocation to specific cellular or biochemical fractions is disrupted. Our analytical method enabled fractionation of the corpus callosum to TBS-soluble and -insoluble fractions^15^ and our results for an effect of cuprizone on copper in the TBS-soluble fraction but not the TBS-insoluble fraction (Fig. 3) illustrate that important factors may be missed if the experimental paradigm is insufficiently nuanced.

We further contend that understanding how copper binding agents affect bioavailability and biodistribution of copper *in vivo* is important to help understand whether an administered agent sequesters copper away from a potentially toxic source or delivers it to a site where physiological requirement is unsatiated. Here it is important to note that copper delivery and copper sequestration away from a toxic source are not mutually exclusive.^45^ Such a dual mechanism could occur in the context of Cu^II^(atsm) in the cuprizone mouse model of demyelination, with Cu^II^(atsm) delivering bioavailable copper, then the resultant copper-free atsm ligand sequestering copper away from a potentially toxic cuprizone–copper complex. Detection of a cuprizone–copper complex in the CNS of cuprizone-treated mice and its clearance following treatment with Cu^II^(atsm) would support this. The movement of copper between the administered agents and *in vivo* biological pools using labeled copper isotopes could also be assessed as previously reported whereby copper from orally administered Cu^II^(atsm) was tracked to confirm its incorporation into endogenous cuproproteins in the CNS.^16^

Our data show that treatment with Cu^II^(atsm) increased copper levels in the CNS and improved myelination in the cuprizone model. Recent cell culture results, however, indicate that release of copper from activated astrocytes can cause demyelination.^46^ Extensive evidence exists for activated astrocytes in affected brain regions in the cuprizone model.^47^ Thus, improved myelination through copper delivery within a neural milieu that involves activated astrocytes may seem irreconcilable with activated astrocytes causing demyelination through copper release. Our results show that cuprizone decreased copper levels in the TBS-soluble fraction of the corpus callosum and that Cu^II^(atsm) remedied this. Notably, the effect of Cu^II^(atsm) on brain copper levels was higher for those regions that were most impacted by the cuprizone treatment. Higher accumulation of the signal for copper in disease-affected regions of brains of patients treated with Cu^II^(atsm)^24–27^ is consistent with copper release being amplified by altered redox conditions affecting stability of the metal-ligand complex and enhanced intracellular trapping of the liberated copper by copper-binding proteins.^48–50^ We propose that properties of Cu^II^(atsm) that determine selective copper release provide important delineation from experimental paradigms and treatments that cannot address the nature of this selectivity. Moreover, previous studies have confirmed that in addition to its ability to selectively deliver available copper to the CNS, mitigation of toxicity from reactive nitrogen and reactive oxygen species is consistent with the intact compound's radical trapping properties.^51–53^ These radical trapping properties could additionally prevent reactive astrocyte-mediated toxicity and prevent down-regulation of myelin associated genes where the involvement of nitric oxide is implicated.^46,54^ Improved myelination through copper delivery and mitigation of toxicity through radical trapping are not mutually exclusive mechanisms of action for Cu^II^(atsm).

Further to these considerations of an additional potential mechanism for Cu^II^(atsm) within the CNS, a limitation of our study is that mice were treated concurrently with cuprizone and Cu^II^(atsm). A potential implication herein is that Cu^II^(atsm)-mediated mitigation of the effects of cuprizone on corpus callosum copper and myelin could involve activity at peripheral sites. This possibility is offset to some extent by *in vivo* evidence for Cu^II^(atsm) selectively providing bioavailable copper within the CNS when compared to peripheral tissue (i.e. liver).^19^ Nonetheless, further experimentation that attempts to delineate CNS from peripheral effects is warranted.

## Conclusions

Collectively, *in vivo* data presented herein indicate a role for disrupted copper availability associated with demyelination in the corpus callosum of the cuprizone model under commonly used experimental conditions. Antidotal effects of Cu^II^(atsm) support the potential to preserve CNS myelination when using a treatment that can selectively deliver available copper within the CNS. Clinical and pre-clinical indications for copper availability as a requirement for healthy myelination, together with widespread utilization of the cuprizone model for examination of demyelination, support the need for better understanding of the mechanistic connection between copper and myelin in the CNS and the processes that contribute to demyelination when this connection is compromised.

## Data Availability

The data underlying this article are available in the article.
